# Supplementary material for: Deep segmentation networks predict survival of non-small cell lung cancer
Source: Sci Rep. 2019 Nov 21;9:17286. doi: 10.1038/s41598-019-53461-2 (PMC6872742; doi:10.1038/s41598-019-53461-2)
Supplement: Supplementary file 1 — Extended Data [file 41598_2019_53461_MOESM1_ESM.pdf]

Deep segmentation networks predict survival of non-small cell lung cancer

Stephen Baek[1,2,3,+], Yusen He[1,+], Bryan G. Allen[2], John M. Buatti[2], Brian J. Smith[5], Ling Tong[4], Zhiyu Sun[1], Jia Wu[6], Maximilian Diehn[6], Billy W. Loo[6], Kristin A. Plichta[2], Steven N. Seyedin[2], Maggie Gannon[2], Katherine R. Cabel[2], Yusung Kim[2,*], and Xiaodong Wu[2,3,*]

[1]University of Iowa, Department of Industrial and Systems Engineering, Iowa City, IA 52242, United States
[2]University of Iowa, Department of Radiation Oncology, Iowa City, IA 52242, United States
[3]University of Iowa, Department of Electrical and Computer Engineering, Iowa City, IA 52242, United States
[4]University of Iowa, Department of Business Analytics, Iowa City, IA 52242, United States
[5]University of Iowa, Department of Biostatistics, Iowa City, IA 52242, United States
[6]Stanford University, Stanford Cancer Institute, Palo Alto, CA 94304, United States
[*]{yusung-kim, xiaodong-wu}@uiowa.edu
[+]these authors contributed equally to this work

Extended Data

Extended Data Table 1. Summary statistics of the data set

	Mean	Median	Std. Dev.	Min	Max
Age at Diagnosis	70.34	71.00	10.59	47.00	90.00
Age at SBRT	71.90	72.36	10.58	47.13	90.17
Group Staging	I	II	III	IV	Other*
	37	10	14	25	10
Smoking Status	Non-smoker	Quit	Light smoker	Smoker	
	10	67	1	18	
Survival Status		Type	Alive	Dead	Other**
	2yr	Overall	61	35	0
		Disease-specific	67	25	4
	5yr	Overall	26	48	22
		Disease-specific	58	14	24

*Patients with no staging information.

**Patients unqualified for the survival category due to the insufficient length of follow-up after initial diagnosis.

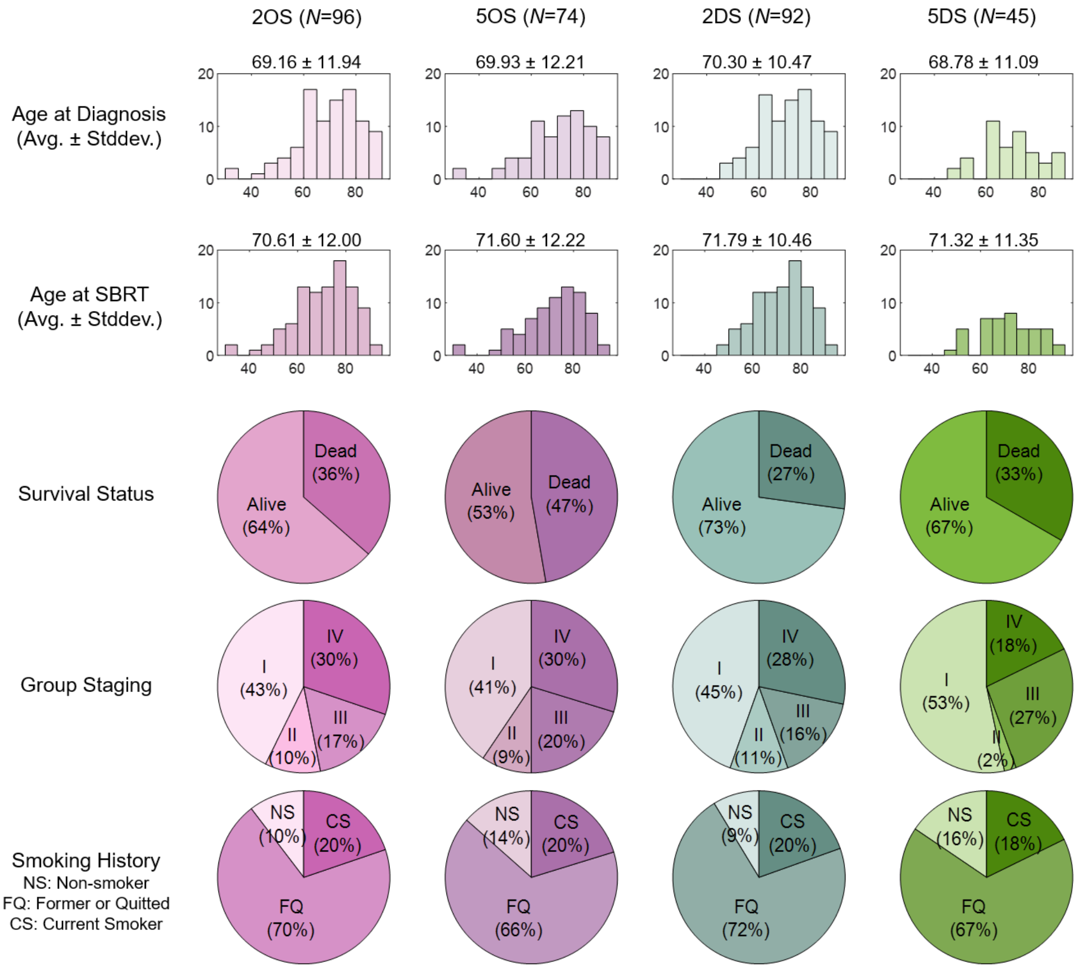

Extended Data Figure 1. Summary statistics of the patients utilized in this study

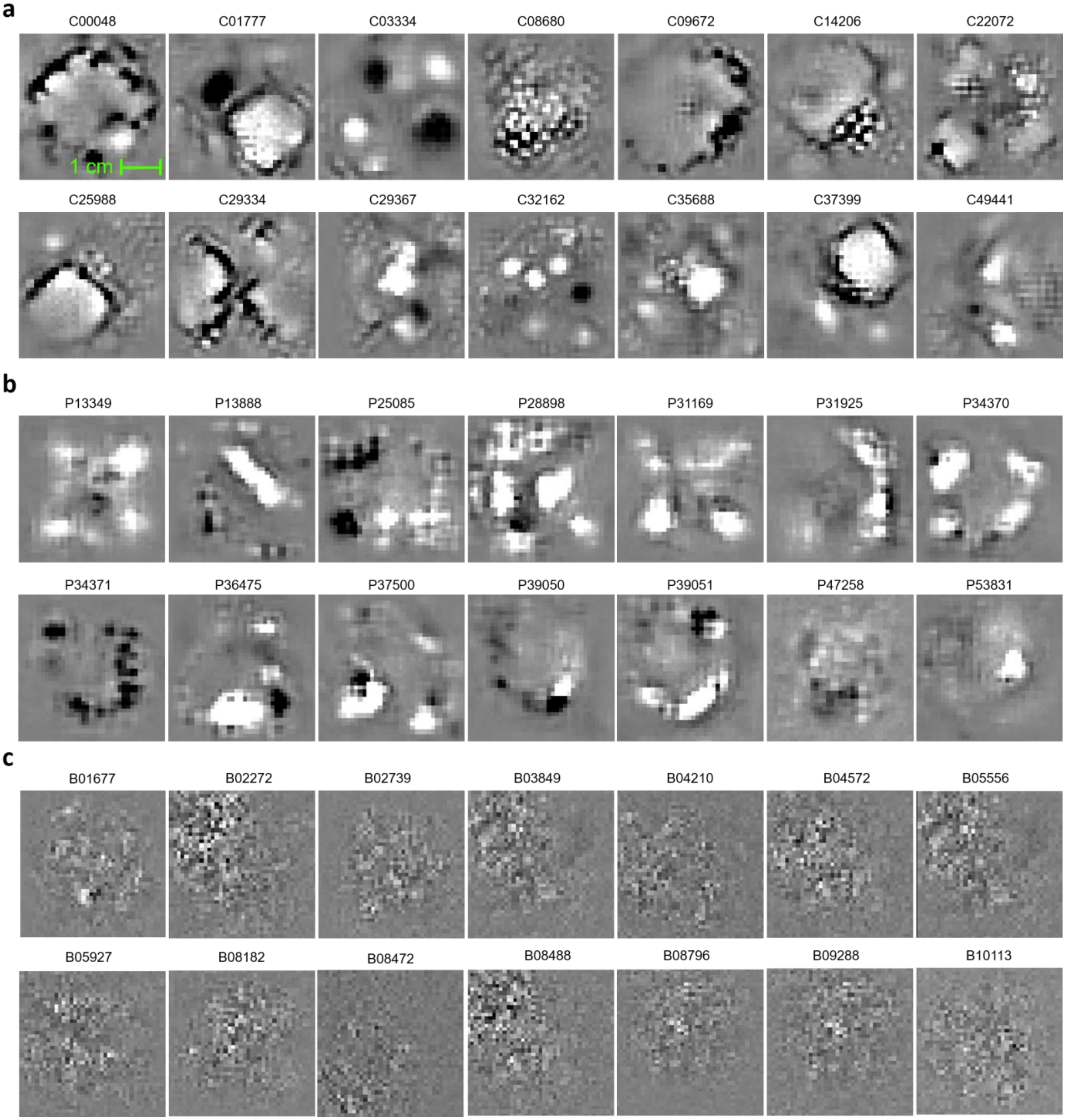

Extended Data Figure 2. Visualization of survival-related features captured by CNNs

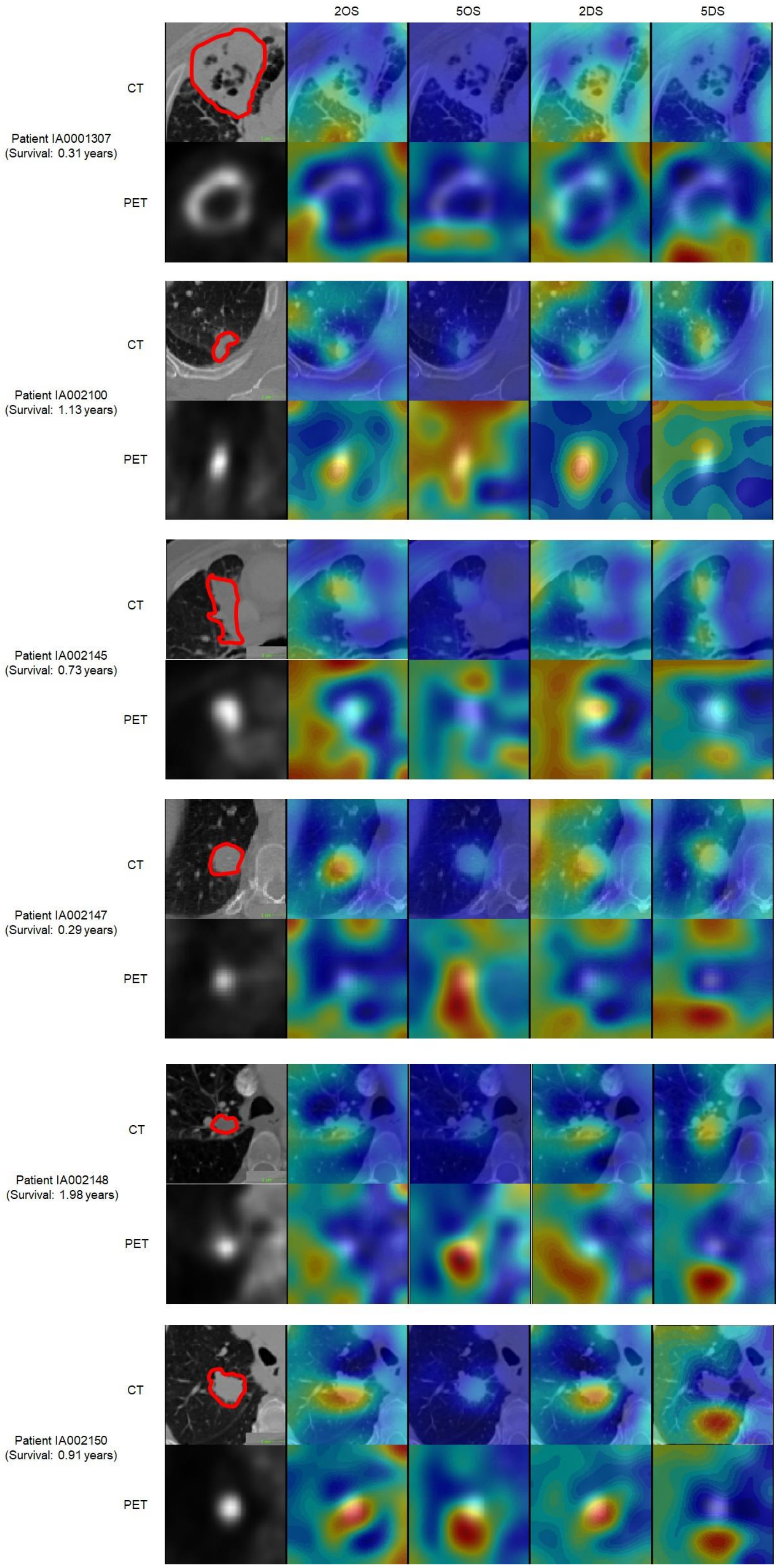

Extended Data Figure 3. Risk map visualization of the patients under 2 years of survival.

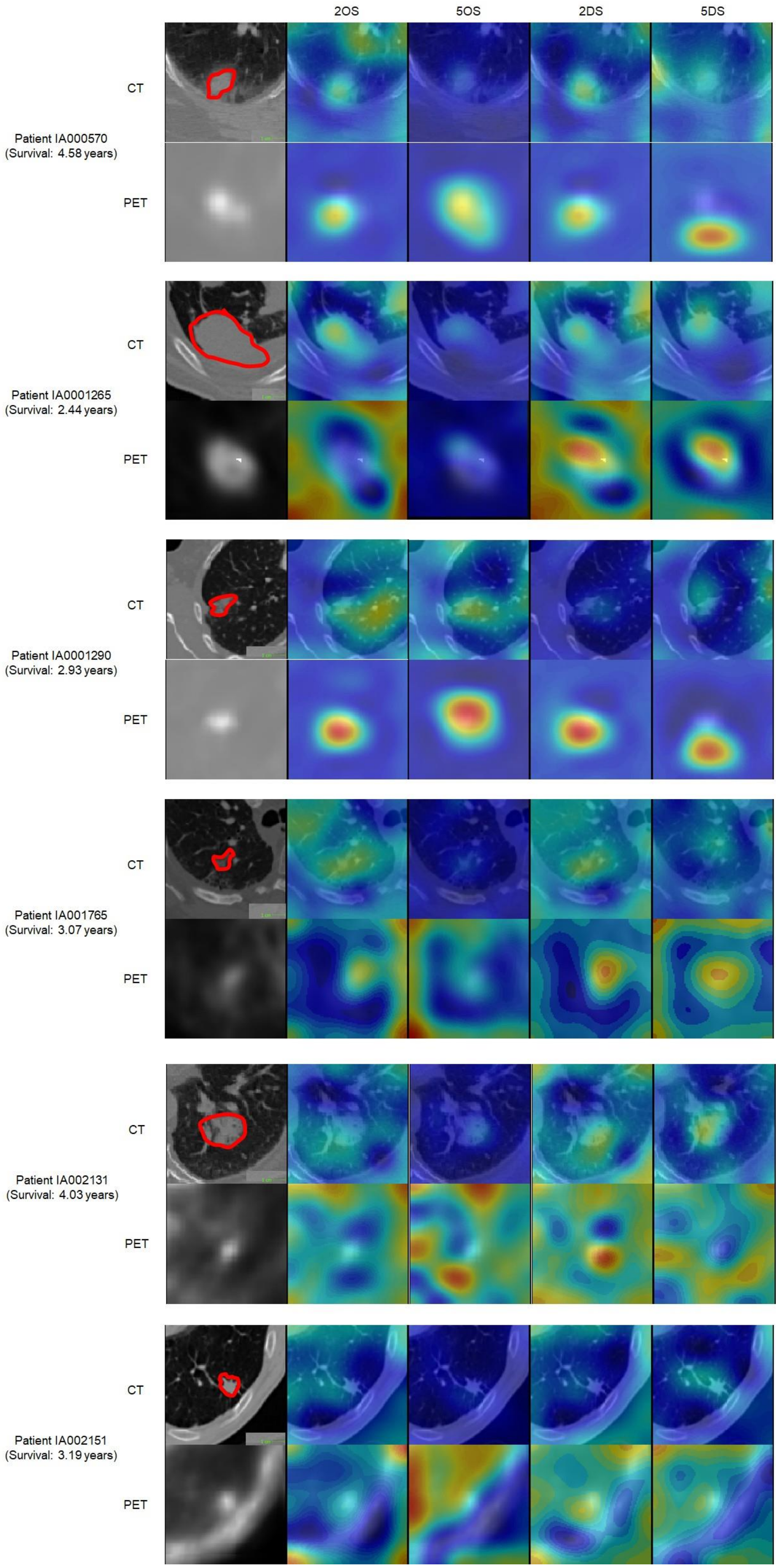

Extended Data Figure 4. Risk map visualization of the patients between 2 and 5 years of survival.

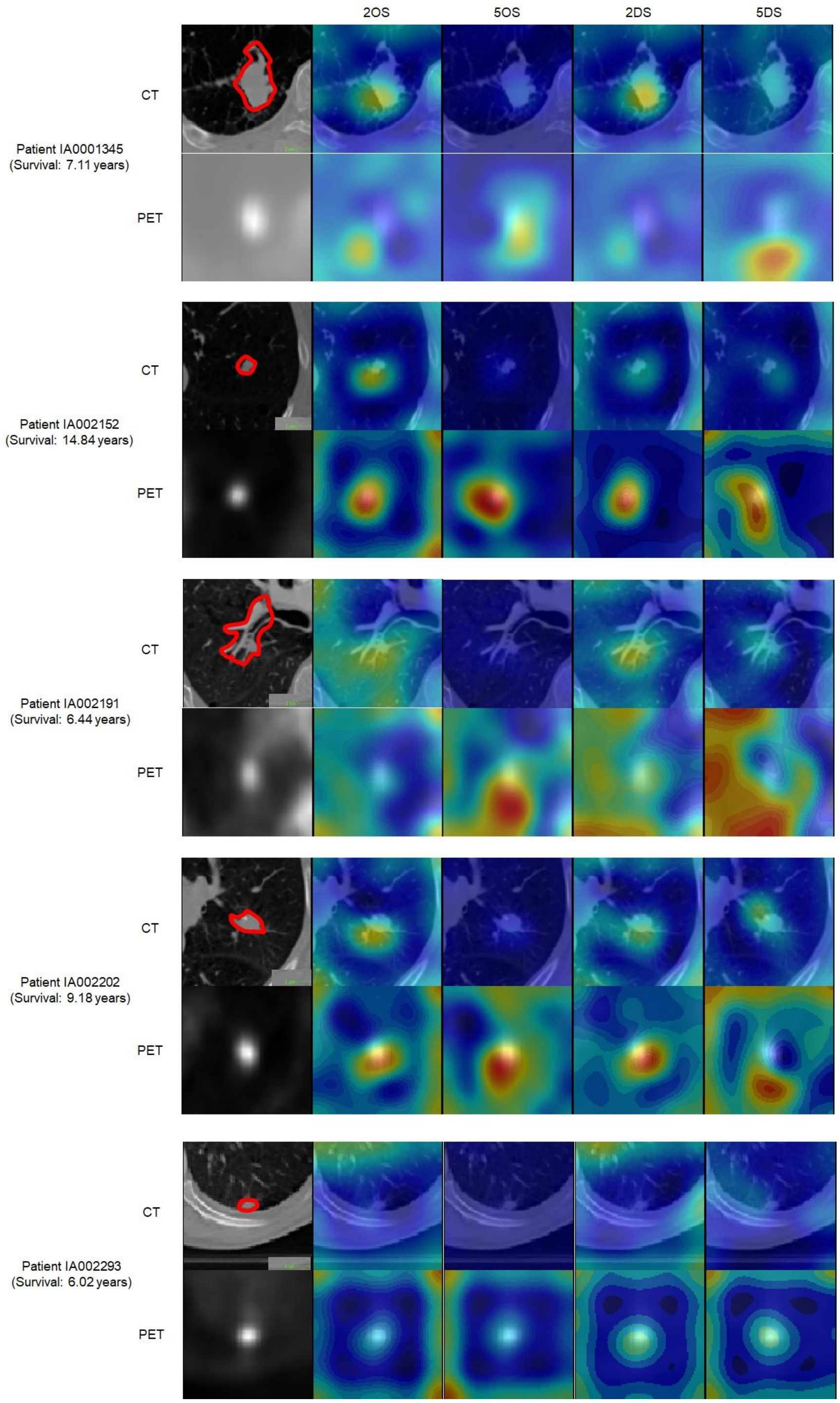

Extended Data Figure 5. Risk map visualization of the patients over 5 years of survival.

Extended Data Table 2. Predictive power of linear model on 2-yr./5-yr. survival outcome

Features		(**a**) TLG		(**b**) Radiomic[28]		(**c**) Benchmark[27]		(**d**) CT U-Net		(**e**) PET U-Net		(**f**) All U-Net	
		2-yr.	5-yr.	2-yr.	5-yr.	2-yr.	5-yr.	2-yr.	5-yr.	2-yr.	5-yr.	2-yr.	5-yr.
Accuracy (95% CI)	OS	0.53 (0.46-0.60)	0.55 (0.47-0.63)	0.62 (0.54-0.70)	0.65 (0.55-0.75)	0.69 (0.65-0.73)	0.75 (0.70-0.80)	0.84 (0.81-0.87)	0.86 (0.80-0.92)	0.83 (0.79-0.87)	0.87 (0.80-0.94)	0.86 (0.79-0.93)	0.89 (0.85-0.94)
	DS	0.57 (0.54-0.60)	0.57 (0.52-0.62)	0.64 (0.59-0.69)	0.80 (0.75-0.85)	0.73 (0.67-0.80)	0.76 (0.69-0.83)	0.86 (0.80-0.92)	0.89 (0.84-0.94)	0.87 (0.81-0.93)	0.88 (0.83-0.93)	0.88 (0.81-0.95)	0.88 (0.82-0.94)
Sensitivity (95% CI)	OS	0.40 (0.27-0.53)	0.32 (0.20-0.44)	0.44 (0.34-0.54)	0.60 (0.53-0.67)	0.63 (0.57-0.70)	0.69 (0.62-0.76)	0.72 (0.61-0.83)	0.81 (0.76-0.88)	0.75 (0.68-0.82)	0.82 (0.74-0.90)	0.74 (0.67-0.81)	0.82 (0.76-0.88)
	DS	0.17 (0.11-0.23)	0.33 (0.23-0.43)	0.40 (0.31-0.49)	0.91 (0.86-0.96)	0.68 (0.60-0.76)	0.70 (0.65-0.75)	0.77 (0.67-0.87)	0.84 (0.78-0.90)	0.79 (0.70-0.88)	0.85 (0.78-0.92)	0.79 (0.73-0.85)	0.86 (0.80-0.92)
Specificity (95% CI)	OS	0.66 (0.55-0.77)	0.78 (0.71-0.85)	0.80 (0.68-0.92)	0.68 (0.60-0.76)	0.77 (0.70-0.84)	0.80 (0.75-0.85)	0.91 (0.78-0.99)	0.90 (0.83-0.97)	0.90 (0.80-0.99)	0.93 (0.84-0.99)	0.91 (0.84-0.98)	0.92 (0.84-0.99)
	DS	0.97 (0.95-0.99)	0.81 (0.71-0.91)	0.88 (0.82-0.94)	0.70 (0.62-0.78)	0.78 (0.72-0.84)	0.81 (0.76-0.86)	0.92 (0.81-0.99)	0.92 (0.86-0.98)	0.90 (0.85-0.95)	0.91 (0.84-0.98)	0.91 (0.83-0.99)	0.91 (0.86-0.96)
AUC (95% CI)	OS	0.60 (0.46-0.74)	0.63 (0.56-0.70)	0.61 (0.48-0.74)	0.69 (0.60-0.78)	0.70 (0.65-0.75)	0.72 (0.66-0.78)	0.83 (0.75-0.91)	0.85 (0.75-0.95)	0.82 (0.73-0.89)	0.87 (0.79-0.95)	0.88 (0.80-0.96)	0.89 (0.85-0.93)
	DS	0.68 (0.61-0.75)	0.68 (0.60-0.76)	0.64 (0.57-0.71)	0.83 (0.79-0.87)	0.72 (0.67-0.77)	0.73 (0.68-0.78)	0.85 (0.75-0.95)	0.85 (0.77-0.92)	0.86 (0.79-0.93)	0.88 (0.80-0.96)	0.86 (0.81-0.91)	0.88 (0.81-0.95)

Extended Data Table 3. Predictive power on the extramural data set

Features		(**a**) Radiomics*		(**b**)Benchmark[27]		(**c**) CT U-Net	
		2-yr.	5-yr.	2-yr.	5-yr.	2-yr.	5-yr.
Accuracy (95% CI)	OS	0.46 (0.34-0.58)	0.71 (0.62-0.80)	0.6 (0.50-0.70)	0.65 (0.53-0.77)	0.82 (0.77-0.87)	0.87 (0.80-0.94)
Sensitivity (95% CI)	OS	0.38 (0.30-0.46)	0.63 (0.52-0.74)	0.4 (0.33-0.47)	0.45 (0.40-0.50)	0.75 (0.65-0.85)	0.9 (0.80-0.99)
Specificity (95% CI)	OS	0.59 (0.51-0.67)	0.74 (0.67-0.81)	0.83 (0.72-0.94)	0.88 (0.80-0.96)	0.91 (0.87-0.95)	0.85 (0.81-0.89)
AUC (95% CI)	OS	0.49 (0.40-0.58)	0.75 (0.67-0.83)	0.65 (0.57-0.73)	0.71 (0.60-0.82)	0.87 (0.80-0.94)	0.9 (0.82-0.98)

*The extramural data set does not have PET images, hence, only 6 features out of the original 17 features in Oikonomou *et al*.[28] were included.
